# A Thermostable Type I Collagen from Swim Bladder of Silver Carp (*Hypophthalmichthys molitrix*)

**DOI:** 10.3390/md21050280

**Published:** 2023-04-28

**Authors:** Honghui Jiang, Yuanyuan Kong, Lili Song, Jing Liu, Zhihong Wang

**Affiliations:** 1 Tianjin Key Laboratory of Biomaterial Research, Institute of Biomedical Engineering, Chinese Academy of Medical Sciences and Peking Union Medical College, Tianjin 300192, China; 2 Tianjin Enterprise Key Laboratory for Application Research of Hyaluronic Acid, Tianjin 300385, China

**Keywords:** swim bladder, thermal stability, collagen, antioxidant activity, silver carp (*Hypophthalmichthys molitrix*)

## Abstract

As a major component of the extracellular matrix, collagen has been used as a biomaterial for many purposes including tissue engineering. Commercial collagen derived from mammals is associated with a risk of prion diseases and religious restrictions, while fish-derived collagen can avoid such issues. In addition, fish-derived collagen is widely available and low-cost; however, it often suffers from poor thermal stability, which limits its biomedical application. In this study, collagen with a high thermal stability was successfully extracted from the swim bladder of silver carp (*Hypophthalmichthys molitrix*) (SCC). The results demonstrated that it was a type I collagen with high purity and well-preserved triple-helix structure. Amino acid composition assay showed that the amounts of threonine, methionine, isoleucine and phenylalanine in the collagen of swim bladder of silver carp were higher than those of bovine pericardium. After adding salt solution, swim-bladder-derived collagen could form fine and dense collagen fibers. In particular, SCC exhibited a higher thermal denaturation temperature (40.08 °C) compared with collagens from the swim bladder of grass carp (*Ctenopharyngodon idellus*) (GCC, 34.40 °C), bovine pericardium (BPC, 34.47 °C) and mouse tail (MTC, 37.11 °C). Furthermore, SCC also showed DPPH radical scavenging ability and reducing power. These results indicate that SCC presents a promising alternative source of mammalian collagen for pharmaceutical and biomedical applications.

## 1. Introduction

As the most abundant protein in human tissues, collagen is primarily distributed in the skin, tendons and cartilage, and is actively involved in biomechanical and biological processes. Owing to their weak antigenic activity, excellent biocompatibility and biodegradability, collagen-based biomaterials have been extensively used in biomedical research, including drug delivery, tissue engineering, and therapeutic drugs [1]. According to their sources, collagen can typically be divided into natural and recombinant collagen. The former collagen is typically made from mammalian tissues such as rat tails, porcine skin, or bovine tendon. However, mammalian-sourced collagens suffer from variable quality, low purity, risk of prion contamination and religious restrictions [2]. Recombinant collagens are produced using synthetic biology and recombinant technology under controlled laboratory conditions. However, it is still difficult to generalize the assembly of the supramolecular band pattern and biological function of native collagen [3].

In recent years, fish and their processing by-products have gained increasing attention due to their accessibility, low risk of disease transmission, lack of religious restrictions and high collagen yield [4,5]. For example, collagen fibrils from Bester Sturgeon were used to coat culture dishes for cell culture, and the results showed that ordered collagen fibers could guide 3T3-L1, MC3T3-E1, C2C12, and L929 cells to grow in the same direction and further induced early osteogenic differentiation of MC3T3-E1 [6]. In addition, collagen from jellyfish (*Catostylus mosaicus*) outperformed rat tail collagen in promoting MC3T3 adhesion and proliferation [7]. Generally, fish collagen is extracted from fish scales, skin or bones [8]. However, fish scales contain more calcium impurities, and fish skin has a high pigment content and a low thermal denaturation temperature [9], which limits their biomedical application. The main components of the swim bladder are collagen, elastin and polysaccharides. A degraded sulfate glycosaminoglycan (D-SBSG) from the swim bladder of bighead carp has been found to have anticoagulant and anti-inflammatory properties, suggesting that it is a potential immunomodulatory drug component [10]. Besides, many studies have focused on the extraction of antioxidant and anti-aging active peptides from fish bladders and their application studies [11,12,13,14]. Compared with fish skin, scales, and other visceral organs, the imino acid content of collagen from swim bladders is typically higher, which corresponds to a higher thermal denaturation temperature [15]. In addition, fish habitat and body temperature are related to the thermal stability of collagen. Thus, collagen from warm-water fish species is more thermally stable than collagen from cold-water fish species [16]. Silver carp (*Hypophthalmichthys molitrix*) is the main fish species used for economic purposes in China, and its annual output value exceeds 3.8 million tons. During processing, approximately 80% of the byproducts from silver carp are not rationally utilized [17]. Taken together, we speculate that the collagen from swim bladder of silver carp is likely to have high thermal stability.

In this study, we prepared a collagen from the swim bladder of silver carp (SCC) using the pepsin extraction method, and collagens from swim bladder of grass carp (GCC), bovine pericardium (BPC) and mouse tail (MTC) were extracted for comparison. The compositional differences in the isolated collagens were identified by SDS-PAGE, peptide maps, and amino acid composition. Their physicochemical properties were identified by ultraviolet-visible spectroscopic (UV) analysis, Fourier transform infrared spectroscopy (FTIR) analysis as well as circular dichroism (CD) measurement. In addition, the thermal stability of the isolated collagens was confirmed by CD. Finally, the fibril-forming and antioxidant capacities of the isolated collagens were studied.

## 2. Results 

### 2.1. Extraction Yields of Collagens 

The yields (%, *w*/*w*) of SCC, GCC and BPC were calculated based on the dry weight of the materials. The yields of SCC, GCC and BPC were 5.92%, 3.92% and 8.53%, respectively. The yield of collagens from two swim bladders were lower than that from bovine pericardium, indicating that the degree of cross-linking among collagen molecules might be stronger in swim bladders from silver carp and grass carp than in bovine pericardium. Similar results have been reported, including collagens from scales of silver carp (1.5%) [18], as well as swim bladder of Amur sturgeon (16.5%) [19] and catla (61.3%) [20]. Differences in collagen yields may be due to the species, structure and composition of tissue, and the extraction method [8].

### 2.2. SDS-PAGE 

**Figure 1:**
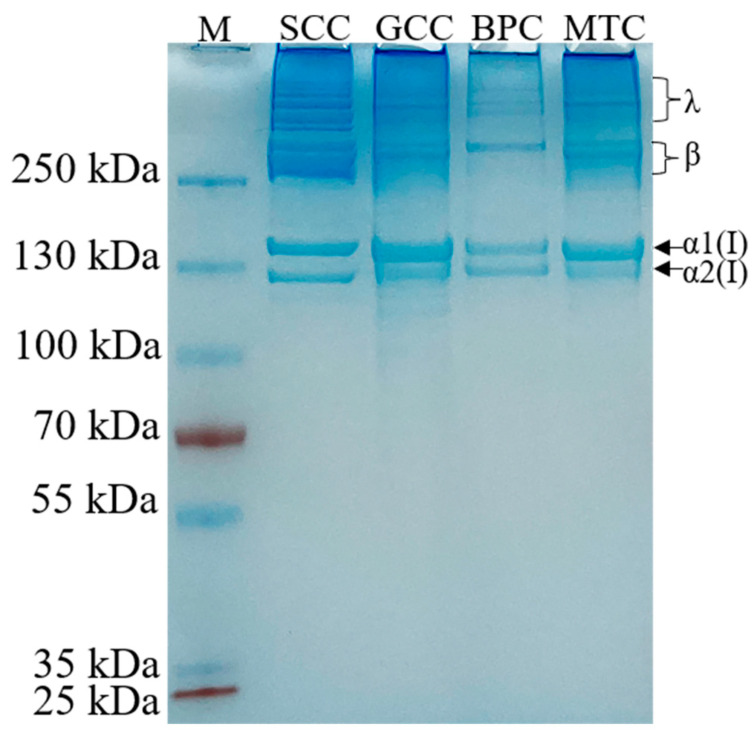
Electrophoretic pattern of collagens from swim bladders of silver carp (SCC) and grass carp (GCC), bovine pericardium (BPC) and mouse tail (MTC). M, marker for protein molecular weight.

All the extracted collagens were identified as type I collagen based on the electrophoretic pattern (Figure 1), which was primarily characterized by two chains (α1(I) and α2(I) with a ratio of roughly 2:1), and their crosslinked chains: β dimers and γ trimers [21]. The molecular weights of α1(I) and α2(I) were approximately 140 and 130 kDa, respectively. Moreover, no other small molecule peptide chains below the α2(I) chain were detected, indicating the high purity of the isolated collagens. Our result was in agreement with those of other type I collagens [22,23]. The electrophoretic patterns revealed that the high molecule crosslinks of collagens were more abundant in SCC than GCC, BPC and MTC, which may be because the cross-linked peptides of SCC are less susceptible to pepsin cleavage.

### 2.3. Peptide Maps

**Figure 2:**
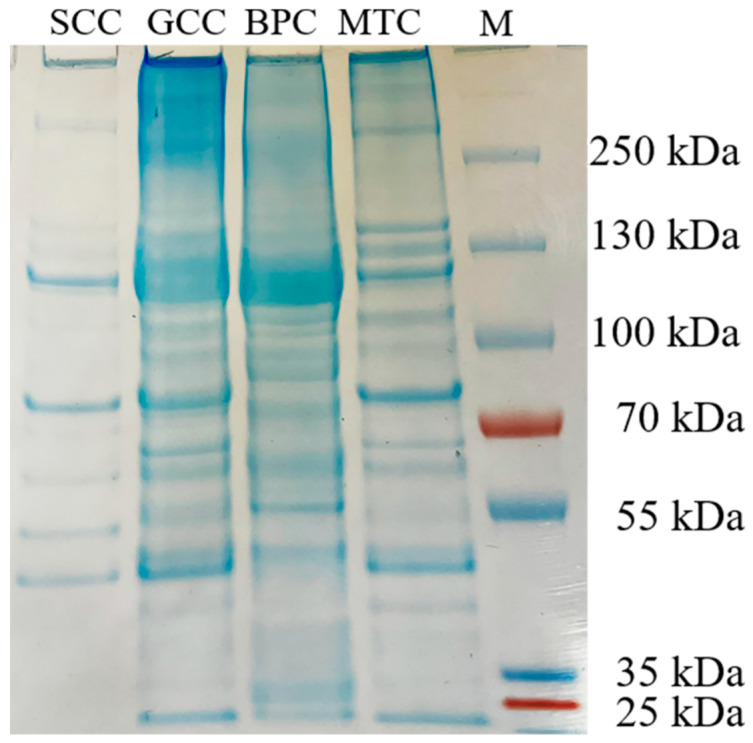
Peptide mapping pattern from collagens from swim bladders of silver carp (SCC) and grass carp (GCC), bovine pericardium (BPC) and mouse tail (MTC). M, marker for protein molecular weight.

The carboxy-terminal peptide chain of hydrophobic amino acids such as tryptophan, tyrosine, phenylalanine, and leucine can be hydrolyzed by chymotrypsin [24]. After chymotrypsin treatment, the densities of the α, β and γ chains decreased, and more bands with low molecular weight were observed (Figure 2). The difference in the band distribution of several collagen-hydrolyzed peptides was mainly distributed at an interval of 55~100 kDa. Thus, differences in peptide mapping suggested some variations in the primary structures of collagens, particularly in amino acid sequence and composition.

### 2.4. Amino Acid Composition

Decellularized extracellular matrix (dECM) is mainly composed of collagen, elastin and polysaccharide, and it has been widely used in tissue engineering and regenerative medicine because of its inherent structure, high bioactivity, low immunogenicity and good biodegradability. Table 1 shows the amino acid composition of the three dECMs and collagens. In three dECMs and collagens, glycine was the most prevalent amino acid, followed by proline, arginine, glutamic acid, and alanine. With the exception of the final 14 residues of the N-terminal amino acid residues and the first 10 residues of the C-terminal amino acid residues, glycine constitutes roughly one-third of all the residues in collagen [25]. Notably, bovine pericardium did not contain cystine, but swim-bladder-derived dECMs and collagens did. In addition, swim-bladder-derived dECMs and collagens had higher concentrations of threonine, methionine, isoleucine, and phenylalanine than bovine pericardium. The pyrrolidine rings found in proline help reinforce the triple helical structure of collagen [26]. The proline content of the collagens extracted in this study is similar to that of the reported collagens with good thermal stability, such as the collagen from swim bladder of rohu (*Labeo rohita*) (145.3 ± 6.28 μg/mg) [15], suggesting that the isolated collagens may have good thermostable abilities. Hydroxyproline can form interchain hydrogen bonds with other amino acids through hydroxyl groups, which are crucial for the thermal stability of collagen [27]. The differences in amino acid contents of collagens and dECMs of SC, GC and BP may be caused by different species. The difference in amino acid content between the dECM and collagen of the same species may be due to the elastin and other proteins contained in dECM.

### 2.5. UV Absorption Spectrum

**Figure 3:**
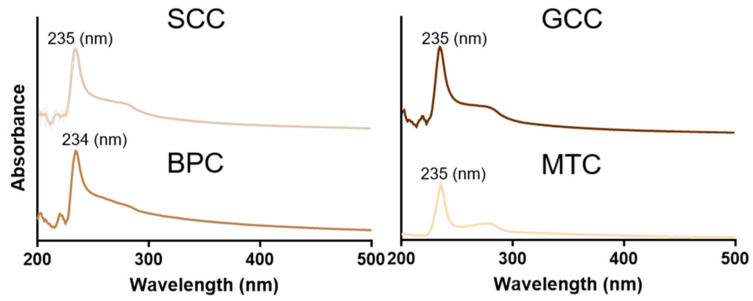
UV spectra of collagens from swim bladders of silver carp (SCC) and grass carp (GCC), bovine pericardium (BPC) and mouse tail (MTC).

Figure 3 showed the UV absorption spectra of the isolated collagens at 200~400 nm. The findings revealed that the maximum absorption peaks of the extracted collagens were all situated between 230 and 240 nm, which was in line with the absorption properties of collagen, similar to that found in carp and barramundi [28,29]. This maximum absorption peak was caused by the n→π* electron leap of C=O, -COOH and CONH_2_. Additionally, SCC, GCC, and MTC all had specific absorption peaks at 260~280 nm, demonstrating the presence of a specific number of aromatic amino acids [17], which corresponds to the results of amino acid composition analysis. It has been suggested that aromatic amino acids, such as tyrosine and phenylalanine, can enhance the antioxidant properties of proteins [30].

### 2.6. FTIR Spectroscopy 

**Figure 4:**
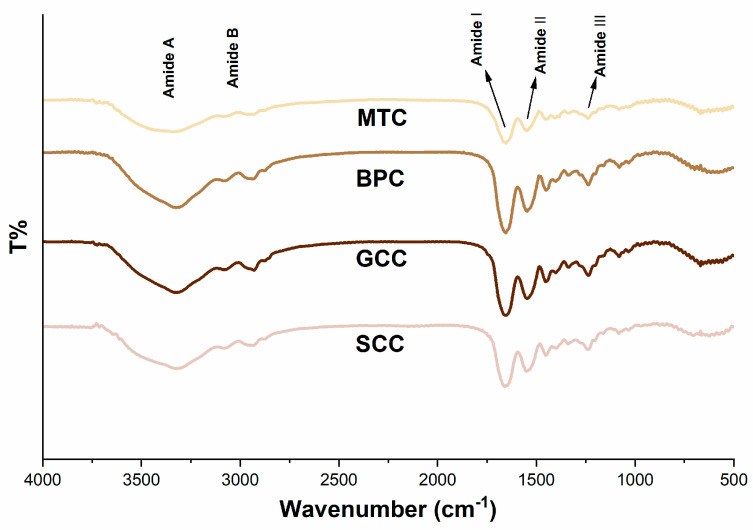
FTIR spectra of collagens from swim bladders of silver carp (SCC) and grass carp (GCC), bovine pericardium (BPC) and mouse tail (MTC).

The FTIR spectra of the isolated collagens demonstrated that they possessed distinctive infrared absorption bands of type I collagen (Figure 4), which are composed of amide A, B, I, II, and III bands [31]. These absorption peaks are caused by the vibration of peptide groups, and can reveal vital details regarding the secondary structure of the protein. In most cases, the amide A band oscillates between 3310 and 3270 cm^−1^, which is correlated with the frequency of the stretching vibration of the O-H and N-H hydrogen bonds. In the present study, the amide A band appeared in the lower wavenumber range, suggesting that the collagen extracted in this study has a certain strength of hydrogen bonding structure [32]. The amide A bands of SCC, GCC, BPC and MTC were 3324.677, 3325.641, 3325.159 and 3338.658 cm^−1^, respectively, indicating that SCC had a higher degree of hydrogen bonding than the other three collagens. Meanwhile, the peak near 2900 cm^−1^ indicates the presence of the amide B band which is mainly produced by the stretching vibration of N–H [33]. An increase in NH–NH_3_^+^ free groups from both lysine residues and the N-terminus is related to the shift of amide B to a higher wavenumber [32]. As seen by the wavenumbers of the amide B bands of SCC, GCC, BPC, and MTC, which were 2936.573, 2930.305, 2935.126, and 2931.751 cm^−1^, respectively, SCC possessed more –NH_3_^+^ free groups than the other three collagens. The amide I band is frequently used as a distinctive indicator of the secondary structure of a peptide. Because the amide I band primarily reflects the C=O stretching vibration and can successfully represent the triple-helix structure, its peak will shift to a lower wavenumber as the molecular order decreases [34]. All of the collagens extracted for this study have a well-preserved triple-helix structure, according to the wavenumbers of the amide I bands of SCC, GCC, BPC, and MTC, which were 1660.41, 1657.035, 1663.302, and 1654.142 cm^−1^, respectively. The amide II band, which is related to the N–H bending vibration of collagen, is present in the wavelength range of 1580 to 1500 cm^−1^ [35], and an amide II band with a lower wavenumber has more hydrogen bonds between N–H groups and a higher degree of structural order. The pyrrolidine ring vibrations of proline and hydroxyproline are related to the N–H bending vibration and C–N stretching vibration of collagen, which are primarily reflected by the amide III band [36]. Our research revealed that the triple helical structures were well-preserved, and that the secondary structures of the isolated collagen were slightly different.

### 2.7. CD Spectrum

**Figure 5:**
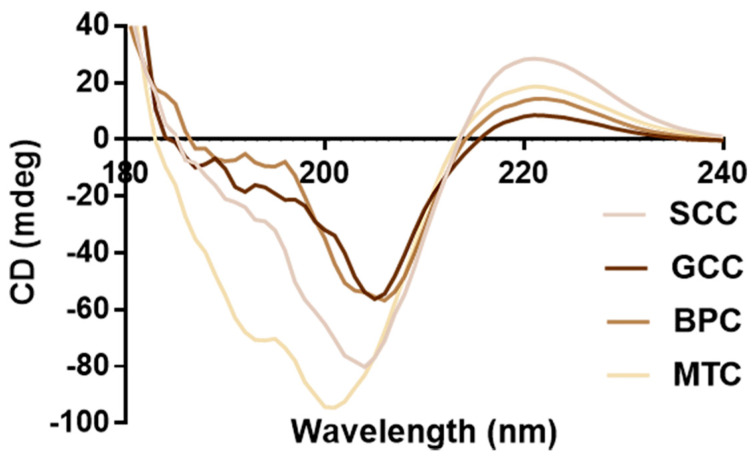
CD spectra of collagens from swim bladders of silver carp (SCC) and grass carp (GCC), bovine pericardium (BPC) and mouse tail (MTC).

Natural collagen has a triple-helix structure that results in a peak that is indicative of the helix at 196~198 nm and a peak that is characteristic of the negative peak at 221~222 nm [37]. Circular dichroism scanning spectra of the four collagens are shown in Figure 5. According to the findings, four collagens showed similar CD spectra line, strong positive absorption peaks close to 221 nm, crossover points on the x-axis at approximately 206 nm, and strong negative peaks at approximately 202 nm. The integrity of the collagen triple-helix structure can be determined by the intensity ratio of the positive and negative peaks (R_pn_) [38]. CD data (Table 2) showed that SCC had the higher CD value at 221 nm (28.6594) and R_pn_ (0.3566) than GCC, BPC and MTC, indicating that collagens had diverse secondary structures. Thus, the triple-helix structures of the extracted collagens were complete, which was in line with the distinctive absorption peaks of the triple-helix structure of collagen in FTIR.

### 2.8. Thermal Stability 

**Figure 6:**
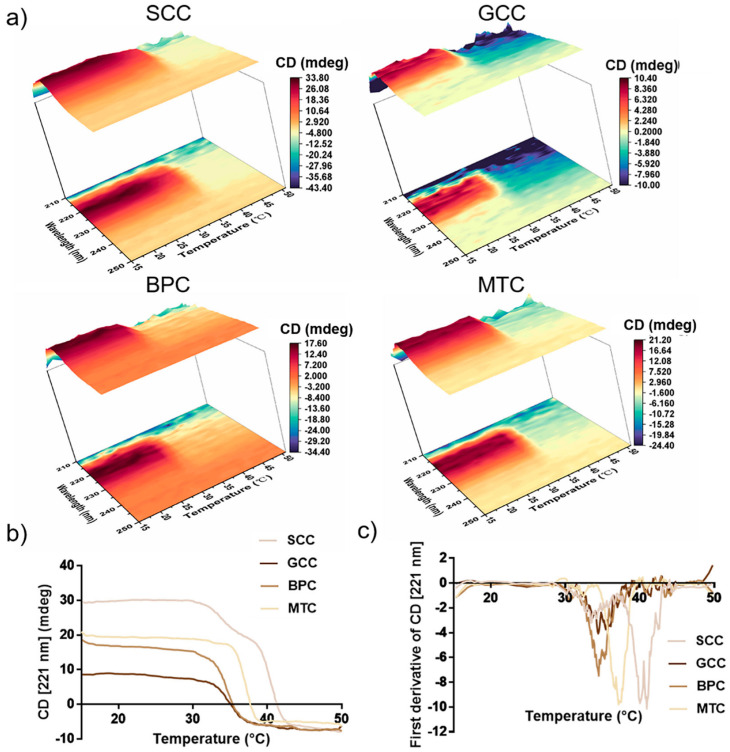
Thermal stability of collagens from swim bladders of silver carp (SCC) and grass carp (GCC), bovine pericardium (BPC) and mouse tail (MTC). (**a**) Thermal denaturation process of the isolated collagens in the wavelength range of 210~250 nm. (**b**) Thermal denaturation curves of four kinds of collagen species at 221 nm and (**c**) differential curve of absorbance of the isolated collagens at 221 nm.

The positive peak in the CD spectra may vanish completely as a result of complete collagen denaturation. In this study, the characteristics of the variation in CD value with temperature for collagen in the wavelength range of 210~250 nm were investigated (Figure 6a). According to our findings, the decrease interval for the CD value of SCC was between 35 and 40 °C, whereas for GCC, BPC, and MTC, it was mainly between 30 and 35 °C. Thus, SCC had higher thermal stability than the other three groups. In order to exactly determine the thermal denaturation temperature of collagens, we examined how the CD value varied with temperature at 221 nm for collagens (Figure 6b,c). The conformational change process of the collagen triple-helix structure is reflected in the variation in the CD value with temperature, thus the characteristic absorbance of the collagen triple-helix structure can be represented by the absorbance (molar ellipticity) at 221 nm in the CD spectrum [39]. Figure 6b showed that as the temperature gradually increased, the CD value of collagen gradually would decrease from a horizontal curve until it stabilized. In our study, the absorbance differential curves of collagen (Figure 6c) were calculated, and the results showed that SCC had the highest denaturation temperature and its differential curve of CD value had two peaks, showing that its denaturation temperature was 40.08 and 40.99 °C, respectively, which may be due to the existence of two collagen molecules with different stability in SCC, or the existence of at least two regions with different stabilities in collagen molecules [40]. In contrast, the absorbance differential curves of GCC, BPC and MTC had single peaks, indicating denaturation temperatures of 34.40, 34.47 and 37.11 °C, respectively. Thus, our results showed that SCC exhibited good thermal stability. 

### 2.9. Collagen Fibrillogenesis

**Figure 7:**
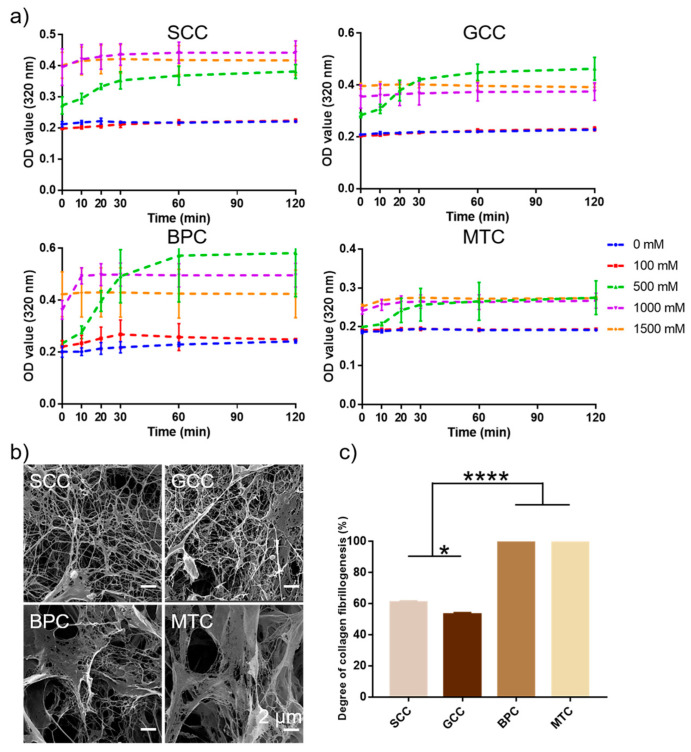
Collagen fibrillogenesis of collagens from swim bladders of silver carp (SCC) and grass carp (GCC), bovine pericardium (BPC) and mouse tail (MTC). (**a**) Self-aggregation kinetics curves of the isolated collagens under different ionic concentration. (**b**) Scanning electron micrographs of collagen fibrils and (**c**) the degree of collagen fibrillogenesis of the isolated collagens under the same conditions. * represents *p* < 0.05, **** represents *p* < 0.0001.

This study looked at the self-aggregation behavior of collagens from four different species sources and compared the effects of different ionic strengths. The findings showed that at a specific ionic strength, the collagen self-aggregation kinetic curves displayed an S-shaped curve (Figure 7a). At ionic strengths of 0 and 100 mM, collagens basically did not self-aggregate, while at ionic strengths higher than 500 mM, collagens could significantly self-aggregate, and the self-aggregation behavior basically reached the equilibrium phase after 1 h. Additionally, with increasing ionic strength (especially 1500 mM), collagen self-aggregation moved more quickly toward equilibrium. In contrast to the collagen fibrils of the bovine pericardium and mouse tail, which appeared as lamellar fibrous structures with larger pore sizes, the collagen fibrils of the two swim bladders were primarily slender fibrous meshwork with slimmer diameters and denser pores (Figure 7b). The degree of self-aggregation of the four collagens varied after 2 h of self-aggregation under the same conditions (Figure 7c), MTC and BPC could reach up to 100% (protein concentration in the supernatant was below the lower limit of detection), whereas SCC and GCC were 60.92% and 53.15%, respectively. The results showed that collagens of mammalian origin performed better in terms of self-aggregation. The different characteristics of collagen fibrils could be the result of varying species [41].

### 2.10. Antioxidant Activity

**Figure 8:**
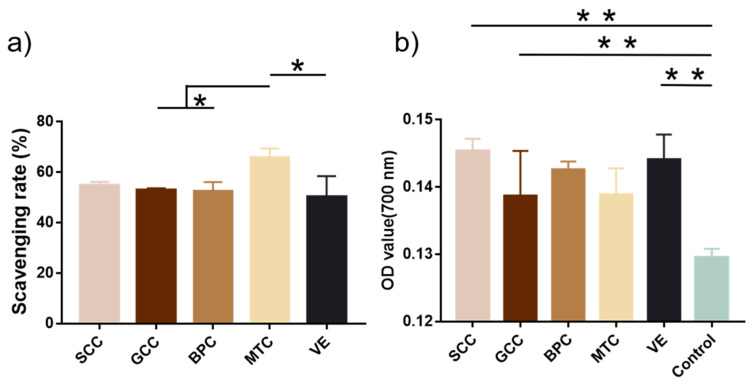
Antioxidant activities of collagens from swim bladders of silver carp (SCC) and grass carp (GCC), bovine pericardium (BPC) and mouse tail (MTC). (**a**) DPPH scavenging rate and (**b**) reducing power of collagens. * represents *p* < 0.05, ** represents *p* < 0.01.

Figure 8a showed that MTC had the highest DPPH radical scavenging rate (65.69%), followed by SCC (54.81%). According to Zhuang et al., collagen and collagen hydrolysate from jellyfish could significantly shield skin lipids and collagen from UV radiation and promote the production of skin collagen [42]. The capacity of collagen to convert iron to ferrous ions determines its reducing power. In terms of reducing ability, SCC was the strongest with an absorbance of 0.145 at 700 nm, followed by that of vitamin E (0.144) (Figure 8b). According to Jeevithan et al., collagen from silvertip shark cartilage exhibited significant DPPH radical scavenging activity and reducing power [31]. 

## 3. Discussion

Owing to its unique properties, collagen is a highly versatile and high-performance biomaterial that is widely used in clinical medicine. The heat denaturation temperature of the protein material should be higher than 37 °C when it is applied to the human body in order to maintain its biological activity. The function of the extracted biomolecules is significantly affected by denaturation because it causes loss of the recognizable triple-helix conformation [43]. Rapid degradation of collagen *in vivo* reduces the effective duration of its biological activity, which is the main limitation of fish collagen. Existing methods usually enhance thermal stability by chemical cross-linking or compounding with other materials, but each method has its own drawbacks, such as the cytotoxicity of glutaraldehyde. Thermal stability of collagen is also directly impacted by the habitat temperatures and the body temperatures of various animal species [44], and SC and GC used in this study were from the same fishery, whose water temperature was approximately 20 °C. The thermal denaturation temperature of the protein can be verified by viscosity test, CD measurement and differential scanning calorimetry. The thermal denaturation temperature of the same collagen obtained using different test methods may vary owing to differences in the methods themselves. Direct detection of the triple-helix structure in collagens can be accomplished using CD measurement, including R_pn_ measurement. In this study, a thermostable type I collagen from swim bladder of silver carp was extracted, and its physicochemical properties were studied. CD measurements showed that the thermal denaturation temperature (T_d_) of SCC was up to 40.08 °C (Figure 5). Besides, SCC had the highest CD value at 221 nm and R_pn_ (Table 2), suggesting a better triple-helix structural integrity. The conformation, amino acid sequence, and imino acid content of collagen play a role in the thermal stability [45]. The proline content of SCC, GCC and BPC were all above 140 μg/mg, similar to the reported thermostable collagens from scale (152.3 μg/mg) [28] and swim bladder (145.3 ± 6.28 μg/mg) [15] of rohu. However, the thermal denaturation temperatures of GCC and BPC were lower than that of SCC, indicating that there are other reasons why these collagen types have differences in thermal stability. We speculate that the following reasons exist for this: 1. Disulfide bonds have an important effect on the stability of tertiary structures, and SCC and GCC contain cystine with disulfide bonds, while BPC does not contain cystine [46], which could be confirmed by the FITR results that the amide A band positions (Figure 4) indicated that the degree of hydrogen bonding interaction was higher for SCC than for the other three collagens. 2. Since serine residues can offer more freedom in the C-N and C-C bonds within the peptide bond than cyclic hydroxyproline residues, they could potentially replace hydroxyproline in collagen. The serine content of SCC, GCC and BPC were 33.35, 37.03 and 32.23 μg/mg, respectively, while the serine content was reported to be negatively correlated with the thermal denaturation temperature [47], which may be one of explanations why GCC has the lowest thermal stability. 

Collagen is a typical amphiphilic molecule containing a large number of hydrophilic amino acids. The hydrated layer of collagen is destroyed by the addition of neutral salts, exposing the charged residues and hydrophobic groups, allowing the monomer collagen molecules to self-aggregate quickly through electrostatic and hydrophobic interactions. Type I collagen can form collagen fibrils by self-aggregation, that is, collagen molecules with a complete triple-helix structure are rearranged in an orderly intermolecular manner to form collagen fibrils with a staggered stripe structure (also known as the D-cycle structure), which can be divided into three processes: 1. Collagen molecules interact with each other to form collagen microfibrils; 2. Collagen microfibrils are assembled into collagen fibers; 3. Equilibrium stage [48]. Collagen fibers are more thermally-stable and resistant to degradation than collagen molecules alone, and can also exist as hydrogels, sponges, or thin films, which can be used in biomaterials and tissue engineering fields. The self-aggregation behavior of collagen is influenced by factors such as the solution pH, ionic strength, temperature, solvent and concentration. In this study, SCC can quickly form a dense fibril structure after adding PB solution containing a certain salt ion concentration, and it has good fibril-forming ability (Figure 7), so its application form and area can be further extended.

The ability of collagens to decrease hydroperoxides, inactivate ROS, chelate pro-oxidative transition metals, eliminate oxidants, and scavenge free radicals is thought to be part of their antioxidant mechanism [49]. The essential quality for cosmeceutical preparations meant to reduce photoaging and UV damage is their radical scavenging activity [50]. The antioxidant properties of proteins are significantly influenced by their size, structure, and amino acid composition [51], thus the high content of hydrophobic amino acids (methionine, isoleucine, and phenylalanine) in SCC may account for its antioxidant properties. In the present study, SCC showed a good ability to scavenge DPPH radicals and reducing power (Figure 8). Ischemic diseases and tumors can cause local tissues in the ROS environment, so further application studies of SCC are necessary in the treatment of these diseases.

There are some limitations in this study, that is, its biological activities, such as the influence on cell proliferation and differentiation and the ability to protect cells in an oxidative emergency environment, were not verified at the cellular level. In the future, SCC can be used to prepare into sheets and injectable materials for further applications.

## 4. Materials and Methods

### 4.1. Materials and Reagents

Fresh swim bladders from silver carp and grass carp were purchased from a local farm, and fresh bovine pericardium was collected from Fuhua Meat Co., Ltd. (Hebei, China). All chemicals were of analytical grade. Acetic acid (AC) was purchased from DAMAO Chemical Reagent Factory (Tianjin, China). NaCl and NaOH were purchased from Sigma Aldrich (Milwaukee, WI, USA). Porcine pepsin was purchase from Shanghai Yuanye Bio-Tech Co., Ltd. (Shanghai, China).

### 4.2. Isolation and Purification of Collagen

Swim bladders and bovine pericardium were rinsed twice with chilled distilled water immediately, cut into pieces, and stored at −80 °C. In order to eliminate pigments and non-collagenous proteins, the frozen materials were combined with 0.15 M NaOH and stirred for 6 h. The washed mixture was defatted with 10% isopropyl alcohol for 24 h and rinsed with chilled distilled water to remove any residual reagent. To extract the collagens, pretreated swim bladders and bovine pericardium were soaked for 48 h in 0.5 M AC with 0.2% (*w*/*v*) porcine pepsin (1:30,000). Then, 0.9 M NaCl was added to the AC solution containing the extracted collagen to salt-precipitate collagen. To ensure the unwanted protein was removed, the salt-precipitating extraction procedure was carried out three times. Collagen was reconstituted in 0.5 M AC, dialyzed for 24 h against 0.02 M AC, and then for 48 h against distilled water using dialysis bags (14 K MWCO, Solarbi, Beijing, China). Following that, the purified collagen was lyophilized for 48 h using a freeze dryer (SCIENTZ-10N, SCIENTZ, Ningbo, China), and then stored at −20 °C. The extraction method of collagen from C57 mouse tail is similar to the above steps, except that the extraction solution is 0.5 M AC without pepsin. dECMs were prepared as described in our previous paper [52]. Briefly, fresh swim bladders and bovine pericardium were firstly treated with sodium dodecyl sulfonate and Triton X-100 with gentle shaking, rinsed with PBS for several days, and then incubated with DNase and RNase overnight.

### 4.3. SDS–Polyacrylamide Gel Electrophoresis (SDS-PAGE)

Lyophilized collagens were solubilized in 0.5 M AC at 2 mg/mL, combined with sample buffer solution, and boiled for 10 min to completely denature proteins. Then, 15 μL of samples was infused onto an 8% polyacrylamide gel for electrophoresis for 30 min at 120 V. The gel was then stained with Coomassie Brilliant Blue staining solution (Biosharp, Hefei, China) and excess stain was removed using deionized water with gentle stirring.

### 4.4. Peptide Maps

Lyophilized collagens (1 mg) were mixed with 0.1 mL of 0.05 M Tris-HCl (pH 7.5, containing 10 mM CaCl_2_) before incubation at 50 °C for 1 h. After that, the mixture received a 10 μL addition of chymotrypsin solution (0.1 mg/mL) for 5 min. The digestion was stopped by adding 130 μL of 5% (*w*/*v*) SDS and boiling at 85 °C for 10 min. The peptides produced by digestion were separated using SDS-PAGE.

### 4.5. Amino Acid Analysis

A high-resolution mass spectrometer (Q Exactive, Thermo, Waltham, MA, USA) and ultra-high performance liquid chromatography (UPLC, Thermo, USA) were used for quantitative analysis to determine the amino acid composition of collagen. Complete hydrolysis of lyophilized collagens with 6 M HCl was performed at 110 °C for 24 h. After that, samples were centrifuged for 10 min at 15,000× *g*. The mixture was then given the AccQ·Tag Ultra Borate buffer after the supernatant had been neutralized with 2 M NaOH. The sample was then added in a UPLC vial along with Borate buffer and AccQ·Tag reagent. The mixture was boiled at 55 °C for 10 min after being kept at room temperature for 1 min. After cooling, 1 μL was then injected. In order to analyze the sample extracts, a UPLC-Orbitrap-MS system (UPLC, Vanquish, MS, QE) was used. Based on individual retention time of each amino acid, the peaks were determined. The amount was determined using the peak areas of the samples and the known concentrations of the standard amino acid mixtures. The μg/mg protein equivalent of each amino acid was then calculated.

### 4.6. Ultraviolet-Visible Spectroscopic Analysis

Lyophilized collagens were solubilized in 0.5 M AC at 2 mg/mL. UV spectra were obtained using a spectrophotometer (Lambda35, PerkinElmer, Waltham, MA, USA) in the 190~400 nm range at 1 nm interval. A 0.5 M AC baseline was established.

### 4.7. Fourier Transform Infrared Spectroscopic Analysis

Lyophilized collagens were combined with KBr at a ratio of 1:100 (*w*/*w*) before being squeezed into a tablet. All spectra were collected using an FTIR spectrophotometer (NICOLET Is10, Thermo, Waltham, MA, USA) at 4000~400 cm^−1^ wavenumber with 4 cm^−1^ resolution.

### 4.8. Circular Dichroism Measurement

A spectrometer (J-750, JASCO, Tokyo, Japan) equipped with a temperature control system was used to perform CD spectroscopy of the collagen samples to determine their molecular conformation and denaturation temperature (T_d_). Lyophilized collagens were solubilized in 0.5 M AC at 0.1 mg/mL and placed into a quartz cell with a 1 mm path length. Then, CD spectra were obtained at 190~250 nm with 0.1 mm interval and a scan rate of 50 nm/min. 

To determine T_d_, in the temperature range of 15 to 50 °C, the ellipticity at 221 nm was observed at a scan speed of 5 °C/min at 210~250 nm wavelength or 1 °C/min at 221 nm. The temperature at which the CD[221] value decreased at the fastest rate, T_d_, was determined to be the temperature where the native-fold and fully unfolded structure met in the middle. A 0.5 M AC baseline was established.

### 4.9. Collagen Fibrillogenesis In Vitro

Lyophilized collagens (*n* = 3 for each group) were solubilized in 0.5 M AC at 2 mg/mL. Samples were mixed with 0.1 M PB solution (pH = 7.0) with a certain concentration of NaCl at a ratio of 1:2 (*v:v*) and incubated for a period of time at 37 °C. The absorbance at 320 nm was obtained at various incubation time using a UV/Vis spectrophotometer (Varioskan Flash3001, Thermo, Waltham, MA, USA).

### 4.10. Measurement of Degree of Collagen Fibrillogenesis

Lyophilized collagens (*n* = 3 for each group) were solubilized in 0.5 M AC at 2 mg/mL. Samples were mixed with 0.1 M PB solution (pH = 7.0) with 1 M NaCl at a ratio of 1:2 (*v:v*). The mixture was incubated for 3 h at 37 °C. During the collagen fibrillogenesis, the clear liquid would turn white. To precipitate collagen fibrils, the solution was centrifuged at 20,000× *g* for 20 min. A BCA kit (Beyotime, Shanghai, China) was utilized to measure the protein content of the supernatant. The degree of collagen fibrillogenesis was defined as follows:

Degree of collagen fibrillogenesis (%) = (C_o_ − C_s_) × 100%/C_o_ (1)

where C_o_ denotes the protein content of the collagen sample before collagen fibrillogenesis, and C_s_ denotes the protein content of the supernatant after collagen fibrillogenesis.

### 4.11. Scanning Electron Microscopy (SEM) Observation

Collagen fibrils were formed as described in Section 4.10, fixed with 2.5% (*v*/*v*) glutaraldehyde in 0.1 M PB solution (pH = 7.0) for 3 h, and then rinsed with PB. After gradient dehydration using ethanol solutions, collagen fibrils were dried using a freeze-drying device and coated with gold using a coater (Oxford Quorum SC7620, Laughton, UK). The microstructures of collagen fibrils were investigated using a SEM (TESCAN MIRA LMS, Brno, Czech Republic) at 3 kV.

### 4.12. Antioxidant Activity

#### 4.12.1. DPPH(2,2-diphenyl-1-picrylhydrazyl) Radical Scavenging Activities

The DPPH radical scavenging activity of collagens was conducted according to a previous reported method [53]. Collagen solution (2 mg/mL, *n* = 3 for each group) was mixed with an equal volume of DPPH-ethanol solution (0.1 mmol/L), incubated for 30 min at 25 °C and centrifugated for 10 min at 1500× *g*. A UV/Vis spectrophotometer was utilized to obtain the absorbance at 517 nm of each 100 μL of supernatant. DPPH radical scavenging activity was defined as follows:

DPPH radical scavenging percentage (%) = (1 − (A_s_ − A_o_)/A) × 100% (2)

where A_s_ is the sample absorbance, A_o_ is the sample blank absorbance and A is the control absorbance. Vitamin E was utilized as the positive control.

#### 4.12.2. Reducing Power

The reducing power of collagens was determined according to a previous reported method [54]. 1 mL of collagen solutions (2 mg/mL, *n* = 3 for each group) were combined with 2.5 mL of 0.2 M PB solution (pH = 7.4) and 2.5 mL of 1% potassium ferricyanide. After that, the sample was incubated for 20 min at 50 °C. After adding 2.5 mL of 10% trichloro AC, the sample was centrifuged at 4500 rpm for 10 min. The supernatant (1 mL) was combined with deionized water (1 mL), and 0.1% ferric chloride (0.2 mL). After reacting for 5 min, the absorbance at 700 nm was measured using a UV/Vis spectrophotometer. Vitamin E was used as a positive control.

### 4.13. Statistical Analysis

Quantitative experiments were conducted at least 3 times. Data were expressed as the mean ± standard deviation (SD). The one-way analysis of variance (ANOVA) from GraphPad Prism software (version 7, GraphPad Software, San Diego, CA, USA) was used to compare the differences. *p* < 0.05 was considered statistically significant, i.e., * represents *p* < 0.05, ** represents *p* < 0.01.

## 5. Conclusions

In this study, we successfully extracted a thermostable collagen from the swim bladder of silver carp. SCC is a type I collagen with high purity and well-preserved triple-helix structure. Compared with GCC, BPC and MTC, SCC exhibited difference on electrophoretic patterns, peptide maps, amino acid composition and biochemical properties. SCC can self-aggregate into fine and dense fibrils under certain conditions and shows well-defined protective ability against oxidation. Notably, T_d_ of SCC could reach up to 40.08 °C, indicating that SCC will be well applied as a promising alternative of mammalian collagen for pharmaceutical and biomedical applications in human.

## Figures and Tables

**Table 1:** Amino acids composition from dECMs and isolated collagens (μg/mg). SC, swim bladder of silver carp; GC, swim bladder of grass carp; BP, bovine pericardium.

Amino Acid	Collagens			dECMs		
	SC	GC	BP	SC	GC	BP
Histidine	4.06	4.22	4.10	5.93	5.53	3.89
Hydroxyproline	65.81	68.96	72.77	47.60	33.08	66.64
Arginine	89.23	95.38	94.62	83.80	75.22	85.29
Serine	33.35	37.03	32.23	34.92	36.43	29.80
Glycine	196.44	204.50	198.35	173.50	173.12	180.46
Aspartic acid	76.61	76.89	72.57	83.61	85.28	65.70
Glutamic acid	108.81	110.83	108.05	104.54	94.06	96.20
Threonine	28.86	29.60	18.61	34.05	40.48	16.58
Alanine	97.75	103.20	101.48	88.76	84.67	90.75
Proline	142.87	149.21	155.08	124.37	113.37	136.70
Lysine	16.92	18.39	17.04	14.62	13.33	12.72
Cystine	1.33	1.13	0.00	5.45	5.08	0.00
Methionine	15.24	11.37	5.01	17.00	12.97	4.90
Tyrosine	5.78	7.31	4.09	27.81	49.44	5.64
Valine	16.77	19.52	19.41	23.60	28.96	17.73
Isoleucine	14.06	15.93	13.16	18.92	21.90	11.76
Leucine	29.36	32.53	27.22	41.50	55.53	25.29
Phenylalanine	23.78	24.37	19.78	25.94	23.88	18.91
Tryptophan	0.45	0.07	0.04	0.02	0.02	0.07

**Table 2:** CD data of collagens from swim bladders of silver carp (SCC) and grass carp (GCC), bovine pericardium (BPC) and mouse tail (MTC).

	CD [221 nm] (mdeg)	R_pn_
SCC	28.6594	0.3566
GCC	8.75611	0.1530
BPC	14.4292	0.2533
MTC	18.7925	0.1958

R_pn_ represents the intensity ratio of positive peak over negative peak (absolute values).

## Data Availability

The data presented in this study are available on request from the corresponding author.
